# Merging bound states in the continuum by harnessing higher-order topological charges

**DOI:** 10.1038/s41377-022-00923-4

**Published:** 2022-07-19

**Authors:** Meng Kang, Li Mao, Shunping Zhang, Meng Xiao, Hongxing Xu, Che Ting Chan

**Affiliations:** 1 School of Physics and Technology, and Key Laboratory of Artificial Micro- and Nano-structures of Ministry of Education, Wuhan University, Wuhan, 430072 China; 2 Department of Physics, The Hong Kong University of Science and Technology, Hong Kong, China; 3 Wuhan Institute of Quantum Technology, Wuhan, 430206 China; 4 School of Microelectronics, Wuhan University, Wuhan, 430072 China

**Keywords:** Photonic crystals, Nanocavities, Metamaterials

## Abstract

Bound states in the continuum (BICs) can confine light with a theoretically infinite Q factor. However, in practical on-chip resonators, scattering loss caused by inevitable fabrication imperfection leads to finite Q factors due to the coupling of BICs with nearby radiative states. Merging multiple BICs can improve the robustness of BICs against fabrication imperfection by improving the Q factors of nearby states over a broad wavevector range. To date, the studies of merging BICs have been limited to fundamental BICs with topological charges ±1. Here we show the unique advantages of higher-order BICs (those with higher-order topological charges) in constructing merging BICs. Merging multiple BICs with a higher-order BIC can further improve the Q factors compared with those involving only fundamental BICs. In addition, higher-order BICs offer great flexibility in realizing steerable off-Γ merging BICs. A higher-order BIC at Γ can split into a few off-Γ fundamental BICs by reducing the system symmetry. The split BICs can then be tuned to merge with another BIC, *e.g*., an accidental BIC, at an off-Γ point. When the in-plane mirror symmetry is further broken, merging BICs become steerable in the reciprocal space. Merging BICs provide a paradigm to achieve robust ultrahigh-Q resonances, which are important in enhancing nonlinear and quantum effects and improving the performance of optoelectronic devices.

## Abstract (web summary)

Merging BICs with robust Q factors against fabrication imperfections can be further improved by higher-charged BICs. Moreover, higher-charged BICs offer great flexibility in realizing steerable merging BICs.

## Graphical abstract

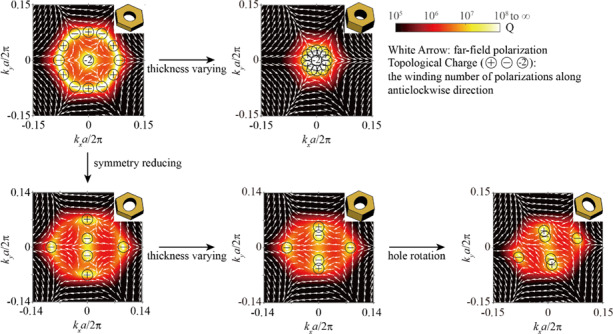

## Introduction

Topological photonics as a burgeoning research field has stimulated extensive studies on various interesting phenomena^1^. While topological physics research usually focuses on near-field phenomena such as topological edge modes, it was recently found that topology also has profound consequences in the far-field phenomena such as the polarization vectors of radiation emerging from photonic crystal slabs (PCSs). Topological notions can explain and predict unusual phenomena including bound states in the continuum (BICs)^2–5^ and circularly polarized states^6,7^. BICs are perfectly confined resonances with theoretically infinite quality (Q) factors, even though their frequencies reside inside the continuous spectrum of radiative states^8^. To date, various mechanisms have been proposed to construct BICs in both quantum^9–12^ and classical waves^2,13–23^. Their unique advantages in trapping light can promote applications in lasing^24–27^, nonlinear optics^28–31^, chemical and biological sensing^32^, metasurfaces^33,34^, optical switches^35^ and vortex beams^36,37^, etc.

In practical applications, inevitable fabrication imperfections will limit the Q factors of BICs due to the coupling with nearby radiative states induced by scattering. To mitigate the scattering loss caused by fabrication imperfections, we can improve the Q factors of the states close to the BICs. A smart way to achieve this goal is using the topological properties of BICs^38^. When $C_2^zT$ symmetry is preserved, BICs on PCSs are topological defects of the polarization vortexes which carry integer topological charges^3^. Fundamental BICs carry topological charges ±1 while higher-order BICs possess topological charges larger than one as allowed by high symmetry groups or on higher energy bands. Topological charge conservation makes fundamental BICs tunable under the variation of structural parameters. Thus, one can tune multiple fundamental BICs to merge at a chosen *k* point. In this way, Q factors of nearby states around the merging BIC can be enhanced over a broad wavevector range^38^. Topological charge conservation also indicates that a higher-order BIC can be regarded as a collection of fundamental BICs, and hence a higher-order BIC itself should also be able to enhance the Q factors of nearby states. It is then natural to ask if the above two mechanisms, i.e., merging BICs and higher-order BICs, can be combined. However, to date, the studies of merging BICs have been limited to fundamental BICs with a topological charge ±1, merging BIC involving higher-order topological charges remains unexplored. Meanwhile, merging BICs are usually formed at high symmetry points such as the Γ point, and off-Γ merging BICs are rare which are useful for applications of BICs requiring angular selectivity^36,39–43^. Though a scheme has been proposed to realize off-Γ merging BICs near the anticrossing of two higher energy bands^44^, schemes for steerable off-Γ merging BICs on an isolated lower energy band are still not available.

In this study, we show that merging multiple BICs with another higher-order BIC can further improve the decay rate of the Q factor away from a BIC. Moreover, higher-order BICs at high symmetric points such as the Γ point can split into a few off-Γ BICs under symmetry reduction, and such a splitting enables new schemes for constructing off-Γ merging BICs on isolated bands as well. We show that merging the BICs split from higher-order BICs and other off-Γ BICs induced by different mechanisms, *e.g*., accidental BICs^2^, enhances the robustness of off-Γ BICs against fabrication imperfections. To be more specific, we consider PCSs exhibiting $C_{6v}$ symmetry, and with such a symmetry, higher-order BICs can be found at the Γ point. Meanwhile, accidental BICs can be generated at off-Γ points owing to the accidental cancellation of radiation. When we vary structural parameters, higher-order BICs are pinned at the Γ point, while accidental BICs can be tuned to merge with the higher-order BICs. In previous work^38^, merging multiple BICs with charges $\pm 1$ has improved the scaling property from $Q \propto k^{ - 2}$ to $Q \propto k^{ - 6}$. Here, merging BICs with a higher-order BIC with a topological charge −2 can further improve the scaling property up to $Q \propto k^{ - 8}$. Because of the improvement of the scaling property, merging multiple BICs in our work has enhanced the Q factors of nearby states by orders of magnitude over a broad range of wavevectors when compared with the Q factors of states near isolated BICs. It has been demonstrated that when the system symmetry is reduced, the higher-order BIC at Γ is split into a few off-Γ BICs^45^. Instead of lattice distortion, we change cylindrical holes from circular to elliptical and similarly the higher-order BIC at Γ is split, and other accidental BICs are preserved at the mirror planes. Under the variation of structural parameters, the off-Γ BICs split from the higher-order BICs can be controlled to remerge with each other at the Γ point or merge with other accidental BICs at off-Γ points. When in-plane mirror symmetry is further broken by rotating the elliptic cylindrical holes, the merging at off-Γ points can occur at any arbitrary point in the reciprocal space.

## Results

**Fig. 1:**
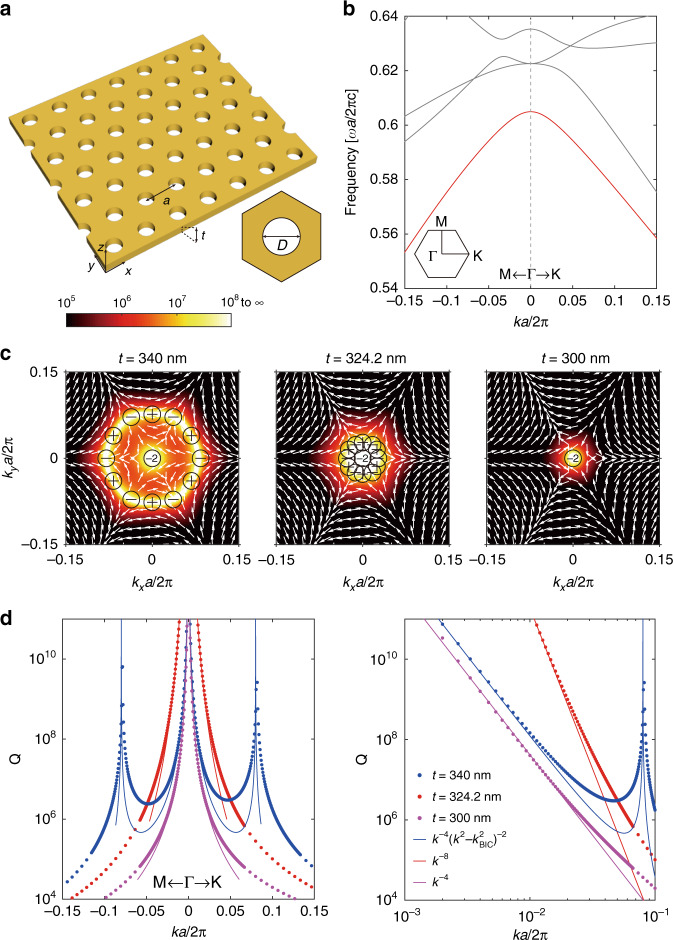
Merging multiple accidental BICs with a higher-order topological charge at Γ. **a** Schematics of a PCS with a triangular lattice of cylindrical holes etched onto a Si_3_N_4_ slab and immersed in a liquid. The lattice constant is *a* = 336 nm and the diameter of the hole is *D* = 160 nm. The inset is the top-view of the unit cell. **b** Simulated TE-like band structure at *t* = 340 nm. The lowest TE-like band is highlighted in red. The inset shows the first Brillouin zone. **c** Simulated polarization vectors around the BICs with the Q factors as the background color for the lowest TE-like band at different thicknesses. When *t* is tuned from 340 nm to 300 nm, a chain of BICs with topological charge $\pm 1$ merges into a symmetry-protected BIC with charge −2. **d** Simulated Q factors (dotted lines) and the corresponding fitting curves (solid lines). The right panel shows the scaling rules along the $\Gamma {{{\mathrm{K}}}}$ direction, and the scaling rules are the same along the $\Gamma {{{\mathrm{M}}}}$ direction. Merging BIC (*t* = 324.2 nm) has considerably enhanced the Q factors of nearby states compared with isolated BICs

To demonstrate merging BICs with a higher-order topological charge, we consider PCSs with a triangular lattice computationally using COMSOL Multiphysics^46^. The PCS consists of a Si_3_N_4_ slab (*n* = 2.02) with cylindrical holes etched (see Fig. 1a) and are immersed in a liquid with a refractive index *n* = 1.46 (common in the laboratory). The bands can be classified as TE-like and TM-like, with respectively $E_z = 0$ and $H_z = 0$ at the mirror plane in the *z*-direction. The TE-like band structures are shown in Fig. 1b, where the lowest band we focus on hereafter is marked in red. The lowest TE-like band is inside the light cone while below the diffraction limit, which thus can radiate into the free space through the zero-order diffraction only. Since the mode at Γ belongs to the B_1_ representation of the $C_{6v}^{}$ point group, it exhibits a charge −2 symmetry-protected BIC (Supplementary Section I) with a divergent Q factor, as shown in Fig. 1c. The system also has $C_2^z$ rotation symmetry, time-reversal symmetry (*T*) and up-down mirror symmetry ($\sigma _h$), which altogether ensure that the system also supports accidental BICs^2^. By tuning geometry parameters of the system, accidental BICs can be realized along the high symmetry direction Μ-Γ-Κ. Beside the charge −2 BIC at Γ, a chain of 12 isolated BICs appears surrounding the Γ point at thickness *t* = 340 nm, period *a* = 336 nm and the diameter of the hole *D* = 160 nm as shown in the left panel of Fig. 1c.

We next reveal the topological nature of BICs by calculating the polarization distribution of the far-field radiation. The polarization vector of far-field radiation is defined as the projection of the Bloch wave function onto the plane basis. The vector field forms a vortex in the reciprocal space with BICs located at the center, and this fact has been revealed both theoretically^3^ and experimentally^4,5^. The topological charge (*q*) of the polarization vortex is defined as the winding number of the polarization vectors^3^, *i.e*., $q = \frac{1}{{2\pi }}{\oint}_C {d{{{\mathbf{k}}}} \cdot \nabla _{{{\mathbf{k}}}}\phi \left( {{{\mathbf{k}}}} \right)}$, where *C* is a closed loop with a counterclockwise direction surrounding the vortex center. Here $\phi \left( {{{\mathbf{k}}}} \right) = \arg \left[ {c_x\left( {{{\mathbf{k}}}} \right) + ic_y\left( {{{\mathbf{k}}}} \right)} \right]$ is the angle of the polarization vector at **k**, with the *x* and *y* components denoted by $c_x\left( {{{\mathbf{k}}}} \right)$ and $c_y\left( {{{\mathbf{k}}}} \right)$, respectively. Figure 1c shows polarization vortexes emerge around BICs, and topological charges of the symmetry-protected BIC and accidental BICs are $q = - 2$ and $q = \pm 1$, respectively. The symmetry-protected BIC is fixed at the Γ point, while topologically protected accidental BICs are tunable under the variation of structural parameters. As shown in the middle panel of Fig. 1c, by decreasing the thickness from *t* = 340 nm to *t* = 324.2 nm while maintaining *a* and *D* unchanged, the chain of accidental BICs is tuned to merge with the symmetry-protected BIC. By further decreasing *t*, opposite topological charges annihilate and only a higher-order BIC with topological charge $q = - 2$ remains at *t* = 300 nm as shown in the right panel of Fig. 1c.

The Q factor distribution along with high symmetry directions $\Gamma {{{\mathrm{M}}}}$ and $\Gamma {{{\mathrm{K}}}}$ at different thicknesses are shown in Fig. 1d, where the Q factors of resonance modes near merging BICs (red) have been significantly enhanced over a broad wavevector range compared with those of isolated BICs (blue and magenta). The scaling rule of Q factors in the vicinity of BICs can be theoretically derived from the Taylor series of $c_x\left( {{{\mathbf{k}}}} \right)$ and $c_y\left( {{{\mathbf{k}}}} \right)$, whose coefficients are determined by the constraint of rotation and mirror symmetries^38^ (Supplementary Section II). When the symmetry-protected BIC and accidental BICs coexist at *t* = 340 nm, the Q factor decays roughly as $Q \propto k^{ - 4}\left( {k^2 - k_{{{{\mathrm{BIC}}}}}^2} \right)^{ - 2}$ away from Γ. With the decrease of the thickness, accidental BICs gradually approach each other (Supplementary Section IV) following the scaling rule. After merging, accidental BICs with opposite topological charges annihilate and only the symmetry-protected BIC remains. When the symmetry-protected BIC is the only preserved BIC at *t* = 300 nm, the Q factor decays as $Q \propto k^{ - 4}$ away from Γ. The two scenarios approximatively follow the same scaling rule $Q \propto k^{ - 4}$ in the vicinity of Γ. In contrast, merging BIC has a different scaling rule $Q \propto k^{ - 8}$, which is a special case of $Q \propto k^{ - 4}\left( {k^2 - k_{{{{\mathrm{BIC}}}}}^2} \right)^{ - 2}$ when $k_{{{{\mathrm{BIC}}}}} \to 0$. Owing to such a scaling rule, the Q factors of the resonances close to the merging BIC are orders of magnitude higher than the other two scenarios. Meanwhile, compared with merging BICs realized in a square PCS where the topological charge of the symmetry-protected BIC is $q = 1$ (ref. ^38^), the scaling rule herein has been further improved from $Q \propto k^{ - 6}$ to $Q \propto k^{ - 8}$ in our system. For a more general case, we can prove that merging BICs with a higher-order BIC with a topological charge $q = \pm n$ can enhance the Q factors of nearby states to $Q \propto k^{ - 2n - 4}$ (Supplementary Section II).

**Fig. 2:**
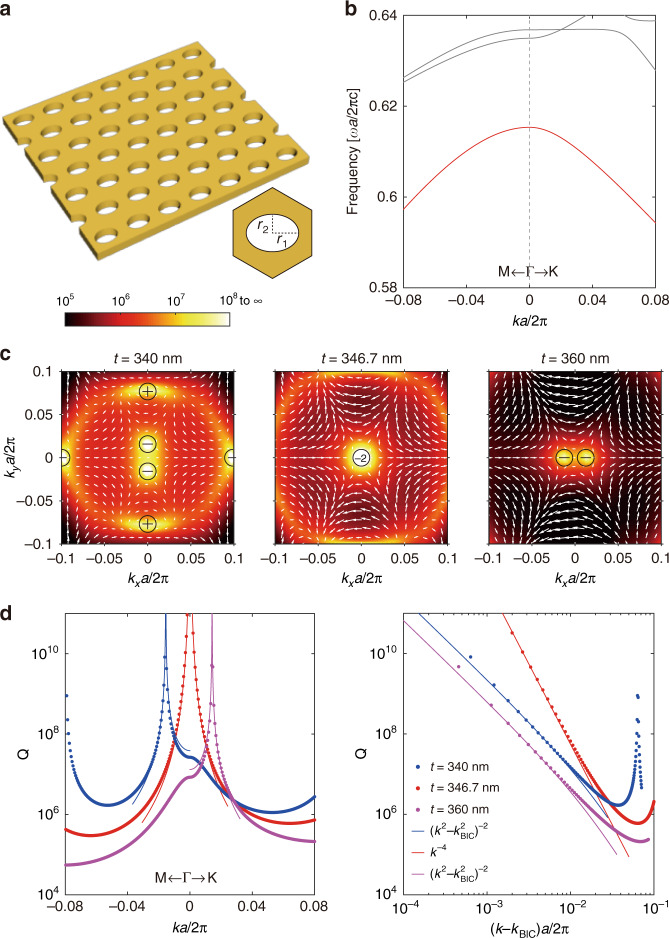
Merging split BICs at Γ. **a** Schematics of a PCS with a triangular lattice (lattice constant *a* = 336 nm) of elliptic cylindrical etched holes. The semi-major axis and semi-minor axis are *r*_1_ = 112 nm and *r*_2_ = 80 nm, respectively. The materials of the PCS and the background are the same as Fig. 1. The inset is the top-view of the unit cell. **b** Simulated TE-like band structure at *t* = 340 nm. The lowest TE-like band is highlighted in red. **c** Simulated polarization vectors around BICs with Q factors as background for the lowest TE-like band at different thicknesses. When *t* is tuned from 340 nm to 360 nm, the two split BICs with the same topological charge −1 bounce off each other. The transition (*t* = 346.7 nm) corresponds to a merging BIC with charge −2. **d** Simulated Q factors (dotted lines) and fitting curves (solid lines). The merging BIC case has considerably enhanced Q factors for nearby states compared with the isolated BICs cases

Next, we break $C_6^z$ symmetry while retaining $C_2^z$ symmetry to generate off-Γ BICs by splitting the symmetry-protected higher-order BIC. As shown in Fig. 2a, elliptic cylindrical holes with the semi-major axis along the *x*-axis are used to implement the symmetry reduction. The TE-like band structures, as shown in Fig. 2b, are similar to the previous ones in Fig. 1b without symmetry reduction except for the lift of band degeneracy at Γ. The lowest TE-like band we focus on is still marked in red, which exhibits a minor distortion under the symmetry reduction (Supplementary Section III). Since the symmetry of the mode at Γ has been reduced to the B_1_ representation of the $C_{2v}$ point group, symmetry-protected BICs are no longer allowed (Supplementary Section I). On the other hand, the topological charge of the higher-order BIC before symmetry reduction cannot suddenly disappear and should split into two separated topological charges at off-Γ points because of the topological charge conservation. The Q factor distribution at *t* = 340 nm is shown in the left panel of Fig. 2c, which indicates that the symmetry-protected BIC is split into two off-Γ BICs when the $C_6^z$ symmetry is broken. Because the system still preserves $\sigma _x$ mirror symmetry, the split BICs are constrained in the ΓΜ direction. The chain of accidental BICs is also distorted by symmetry reduction, with four pairs of positive and negative charged BICs annihilated. As a result, only accidental BICs located in the mirror planes are preserved. The polarization vortex in Fig. 2c indicates that the split BICs both have a topological charge $q = - 1$ (topological charge conservation) and accidental BICs have a topological charge $q = \pm 1$.

Since the system exhibits inversion symmetry, the two split BICs are inversion symmetric with respect to the Γ point. Thus if the split BICs can be tuned back to the Γ point by varying structure parameters, these two split BICs should be back at Γ simultaneously to form a merging BIC. As shown in the middle panel of Fig. 2c, the two split BICs merge at the Γ point when *t* = 346.7 nm. We note that such a merging state is accidental and is different from the symmetry-protected higher-order BICs which are not allowed with only the $C_{{{{\mathrm{2v}}}}}$ point group. Merging BIC has a topological charge $q = - 2$, which is the summation of the topological charges of the two split BICs. When *t* is further increased, merging BIC splits into two isolated BICs along the other mirror-symmetric direction, *i.e*., $\Gamma {\rm K}$, as shown in the right panel of Fig. 2c at *t* = 360 nm. The Q factors of the two split BICs decay in momentum space following the scaling rule $Q \propto \left( {k^2 - k_{{{{\mathrm{BIC}}}}}^2} \right)^{ - 2}$ (Supplementary Section II), while the scaling rule becomes $Q \propto k^{ - 4}$ for the merging BICs, as shown in Fig. 2d. A comparison between the two split BICs and merging BIC confirms that the Q factors of nearby resonances have been enhanced by orders of magnitude for the merging BIC case.

**Fig. 3:**
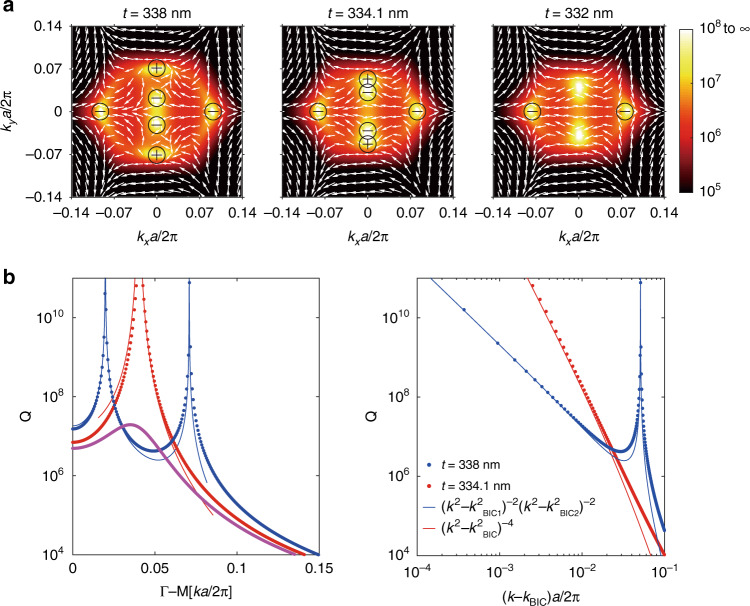
Merging accidental BICs and the split BICs at off-Γ points. **a** Simulated polarization vectors around BICs with Q factors as background at different thicknesses. When *t* is tuned from 338 nm to 332 nm, accidental BICs and the split BICs with opposite topological charges annihilate each other. The transition (*t* = 334.1 nm) corresponds to a merging BIC. **b** Simulated Q factors (dotted lines) and fitting curves (solid lines). The merging BIC case has considerably enhanced Q factors for nearby states compared with the isolated BICs case. In these simulations, we consider the same system as shown in Fig. 2a and only vary *t*

Up to this point, we have investigated the merging of BICs at the Γ point in two different schemes, *i.e*., merging accidental BICs with a higher-order symmetry-protected BIC and re-merging the two split BICs. Merging at high symmetry points such as the Γ point in the reciprocal space is easy to achieve. In contrast, merging BICs at an off-Γ point is quite rare which has only been realized by merging a Friedrich-Wintgen BIC and an accidental BIC^44^. On the other hand, merging at an off-Γ point can also improve the robustness of off-Γ BICs, which are highly desirable for BIC-related applications requiring momentum selection^44^. Here, we propose and demonstrate a new scheme for merging BICs at an off-Γ point by tuning accidental BICs and the split BICs to merge. We still consider the system as shown in Fig. 2a, keeping the period and the shape of the elliptic cylindrical hole unchanged while slightly decreasing the thickness *t*. As shown in the left panel of Fig. 3a, accidental BICs and the split BICs are all located at off-Γ points when *t* = 338 nm. By varying the thickness, the above two different mechanisms induced BICs are tuned to approach each other along the $\Gamma {\rm M}$ direction (Supplementary Section IV). When the thickness is decreased to *t* = 334.1 nm, accidental BICs and the split BICs merge as shown in the middle panel of Fig. 3a. Owing to their opposite topological charges, the two BICs annihilate each other by further decreasing *t* and evolve into a quasi-BIC subsequently as shown in the right panel of Fig. 3a. The Q factor of the quasi-BIC is not infinite but remains pretty large which can be considered as a supercavity^47^. The Q factor distribution in the $\Gamma {\rm M}$ direction is shown in Fig. 3b, indicating that the merging BIC design has significantly larger Q factors over a broad wavevector than either the accidental BICs or the split BICs. The Q factor decays away from BICs following a scaling rule $Q \propto \left( {k^2 - k_{{{{\mathrm{BIC}}}}1}^2} \right)^{ - 2}\left( {k^2 - k_{{{{\mathrm{BIC2}}}}}^2} \right)^{ - 2}$ (Supplementary Section II) at *t* = 338 nm, while the scaling rule becomes $Q \propto \left( {k^2 - k_{{{{\mathrm{BIC}}}}}^2} \right)^{ - 4}$ for the merging BIC at *t* = 334.1 nm. The different scaling rules for the merging BIC can improve the performance of off-Γ BICs in applications.

**Fig. 4:**
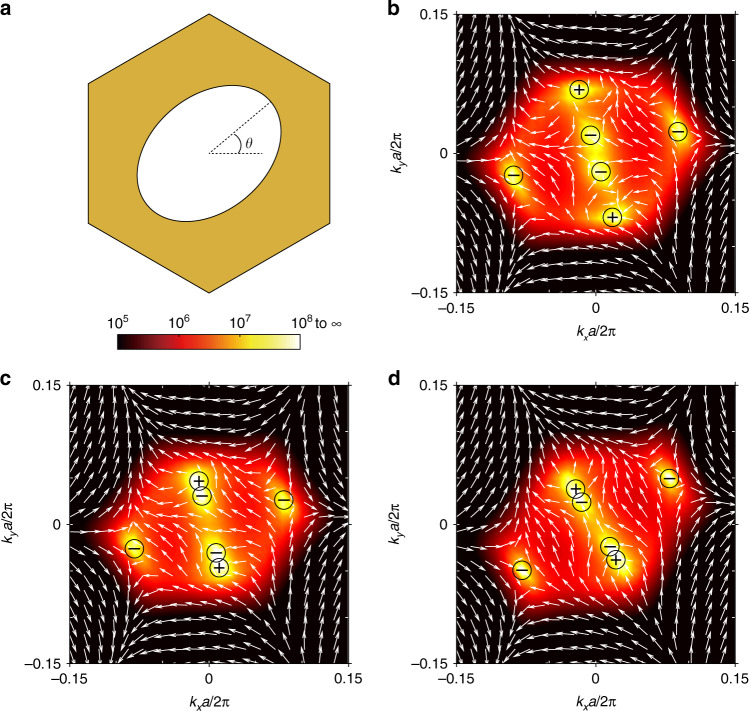
Merging accidental BICs and split BICs at arbitrary k points. **a** The unit cell with elliptic cylindrical etched holes. Calculated distribution of polarization vectors with Q factors as background for **b** isolated BICs with *θ* = 10° and *t* = 338 nm, **c** merging BIC at $( \mp 0.025\pi /a, \pm 0.085\pi /a)$ with *θ* = 10° and *t* = 334.7 nm, and **d** merging BIC at $( \mp 0.045\pi /a, \pm 0.07\pi /a)$ with *θ* = 35° and *t* = 335.6 nm. In **b-d**, the lattice constant, semi-major axis and semi-minor are kept *a*t *a* = 336 nm, *r*_1_ = 110 nm and *r*_2_ = 80 nm, respectively. The materials used are the same as before

Next, we demonstrate that merging BICs can be tuned to an arbitrary point in the reciprocal space by further breaking in-plane mirror symmetries but keeping $C_2^z$ symmetry. BICs are topologically protected when the system maintains both $C_2^zT$ and $\sigma _h$ symmetry^3^, where $\sigma _h$ symmetry ensures the same radiation loss into the up and down subspaces^48^. If $C_2^z$ symmetry is broken, isolated BICs will disappear and become circularly polarized states, and the topological charge $\pm 1$ will split into pairs of half topological charges^7,45,48^. In-plane mirror symmetries constrain BICs to be along with the mirror-symmetric directions. Therefore, we need to break in-plane mirror symmetries to tune both accidental BICs and the split BICs away from the $\Gamma {\rm M}$ direction. This can be done by anticlockwise rotating the elliptic cylindrical hole around the *z*-axis with an angle *θ*, as shown in Fig. 4a, where all the in-plane mirror symmetries are broken while $\sigma _h$ is preserved. As shown in Fig. 4b, isolated BICs are tuned to be away from the $\Gamma {\rm M}$ direction when *θ* = 10°. By decreasing the thickness *t*, accidental BICs are tuned to approach Γ, while the split BICs are tuned to be away from Γ. Thus eventually, the two BICs can be tuned to the same *k* point in the reciprocal space. As shown in Fig. 4c, when *t* = 334.7 nm, accidental BICs and the split BICs merge at $( \mp 0.025\pi /a, \pm 0.085\pi /a)$. When we further decrease *t*, the two BICs annihilate and the polarization vortex disappears as well, which further confirms the existence of a merging BIC. By choosing the appropriate *θ* and *t*, merging BICs can be designed to appear at arbitrary *k* points. For example, as shown in Fig. 4d, merging BICs are tuned to another point $( \mp 0.045\pi /a, \pm 0.07\pi /a)$ when *θ* = 35° and *t* = 335.6 nm.

## Discussion

In summary, we have demonstrated a variety of schemes for merging multiple isolated BICs by manipulating BICs with higher-order topological charges. We start with a PCS in a triangular lattice that allows the cooccurrence of symmetry-protected BICs and accidental BICs. The symmetry-protected BIC with a higher-order charge $q = - 2$ is fixed at the Γ point, while accidental BICs with charge $q = \pm 1$ are tunable in the reciprocal space under the variation of structural parameters. By varying the thickness, we tune multiple accidental BICs to merge with the symmetry-protected BIC at the Γ point. When rotation symmetry $C_6^z$ is reduced to $C_2^z$ using elliptic holes, the symmetry-protected BIC at Γ is split into two off-Γ BICs, and accidental BICs are preserved in mirror planes. The split BICs are then tuned to merge with each other at the Γ point and to merge with another accidental BIC at off-Γ points in the $\Gamma {\rm M}$ direction by varying the thickness. When in-plane mirror symmetries are further broken by rotating the elliptic cylindrical holes, we can realize merging BICs at arbitrary points in the reciprocal space by merging the split BICs and accidental BICs.

We have discussed the merits of BICs with topological charges −2 at Γ, and more flexible schemes can be devised if one considers BICs with topological charges larger than 2 (see, e.g., ref. ^49^). Merging BICs with robust Q factors (Supplementary Section V) can boost light-matter interaction, such as nonlinear and quantum effects, and improve the performance of optoelectronic devices. For example, merging BICs can improve the performance of lasing, nonlinear conversion efficiency and light confinement in zero-index materials. Off-Γ merging BICs have merit in applications requiring momentum selectivity, such as beam steering^36,39^, directional vector beams^40^ and diffraction-free beams^41^, etc. Our work shows that the higher topological charge the involved BICs possess, the more robust the merging BIC is. Meanwhile, higher-order BICs also offers more freedom in forming merging BICs at either high symmetry point such as Γ or other off-Γ points. The Q factor is dominated by the intrinsic loss of materials at merging BICs, and thus materials with ultralow intrinsic loss should be an optimal choice for a superhigh-Q cavity (Supplementary Section VI). We have focused the discussion on the construction of merging BICs on a nondegenerate band TE_1_. Bands TE_2_ and TE_3_ which are degenerate at Γ also support a charge −2 symmetry-protected BIC at Γ and other accidental BICs. We can also extend our schemes discussed above to form merging BICs for degenerate bands (Supplementary Section VII).

## Materials and methods

We perform numerical simulations using COMSOL Multiphysics^46^ in three-dimensional models. The unit cell is hexagonal and consists of a Si_3_N_4_ (*n* = 2.02) slab and an etched hole. The ambient liquid has a refractive index *n* = 1.46. Periodic boundary conditions are imposed in the *x-y* plane. Perfectly matching layers are used in the *z*-direction to absorb any radiation from the PCS. The eigenfrequency solver is implemented to calculate the band structures, eigenmodes ${{{\mathbf{E}}}}\left( {k_x,k_y} \right)$, and Q factors for each $k_x$ and $k_y$. The polarization vector of far-field radiation in the reciprocal space here in our work is defined as

1 $$\begin{array}{ll}{{{\mathbf{c}}}}\left( {k_x,k_y} \right) &= \left( {c_x,c_y,c_z} \right) \\&= \mathop {\iint}\nolimits_{cell} {e^{ik_xx + ik_yy}{{{\mathbf{E}}}}\left( {k_x,k_y} \right)dxdy/\mathop {\iint}\nolimits_{cell} {dxdy} }\end{array}$$

where the integration is performed inside a unit cell of an *x-y* plane slice above the PCS. An alternative approach of defining the polarization vector of far-field radiation using the S and P polarized light basis can be found in ref. ^45^. The topological charge of BIC is defined with the projected polarization vector $\left( {c_x,c_y} \right)$. Stokes parameters are then obtained as $S_0 = \left| {c_x} \right|^2 + \left| {c_y} \right|^2$, $S_1 = \left| {c_x} \right|^2 - \left| {c_y} \right|^2$, $S_2 = 2{\Re} \left( {c_xc_y^ \ast } \right)$, and $S_3 = - 2{\Im} \left( {c_xc_y^ \ast } \right)$. The polarization orientation is defined as $\phi = \frac{1}{2}\arg \left( {S_1 + iS_2} \right)$. When the polarization is linear, it becomes $\phi = \arg \left( {c_x + ic_y} \right)$. The structural systems we study herein all exhibit $C_2^z$ rotation symmetry, which then makes the polarization almost linear^50^ within the interested wave vector range (Supplementary Section VIII).

## Supplementary information

Supplementary information

## Data Availability

The data that support the findings of this study are available from the corresponding authors upon reasonable request.
