# Supplementary material for: Merging bound states in the continuum by harnessing higher-order topological charges
Source: Light Sci Appl. 2022 Jul 19;11:228. doi: 10.1038/s41377-022-00923-4 (PMC9296527; doi:10.1038/s41377-022-00923-4)
Supplement: Supplementary file 1 — Supplementary information [file 41377_2022_923_MOESM1_ESM.pdf]

Supplementary Information for

Merging Bound States in the Continuum by Harnessing Higher-order Topological Charges

Meng Kang[1,2], Li Mao[1,3], Shunping Zhang[1,3], Meng Xiao[1,3,#], Hongxing Xu[1,3,4] and Che Ting Chan[2,†]

[1]*School of Physics and Technology, and Key Laboratory of Artificial Micro- and Nano-structures of Ministry of Education, Wuhan University, Wuhan 430072, China*

[2]*Department of Physics, The Hong Kong University of Science and Technology, Hong Kong, China*

[3]*Wuhan Institute of Quantum Technology, Wuhan 430206, China*

[4]*School of Microelectronics, Wuhan University, Wuhan 430072, China*

Correspondence: #phmxiao@whu.edu.cn, †phchan@ust.hk

This PDF file includes:

I. Allowed topological charges at the Γ point

II. Theoretical models for polarization vortex and scaling rules of Q factor

III. Evolution of band structures

IV. Evolution of BICs with the variation of structural parameters

V. Robustness of merging BICs against fabrication imperfections

VI. Intrinsic loss of materials

VII. Merging BICs in degenerate bands

VIII. Stokes parameters S_3 of polarization vectors

Figs. S1 to S6

References

I. Allowed topological charges at the Γ point

Far-field polarization vectors in the reciprocal space are constrained by the symmetry of the photonic crystal slabs (PCSs). Therefore, the eigenvalue of the rotation symmetry can determine the possible topological charges of the polarization vortexes[1]. Here we focus on the allowed charges of the nondegenerate states at high symmetry points in the reciprocal space. As for the degenerate states, the possible values of charges carried by the degenerate states can be calculated with the eigenvalue of mirror symmetry along with the surrounding mirror-symmetric directions. More details can be found in Ref. [2] and we will not discuss them in detail.

The allowed charges for nondegenerate states exhibiting the corresponding symmetries are summarized in Table S1. The nondegenerate states belong to either A or B representation of the C_{mv} (m = 2, 3, 4, 6) symmetry as given in the second row. A and B representations have C_{m}^{z} rotational eigenvalues +1 and -1, respectively. The eigenvalue of the C_2^z symmetry is also provided in the fourth row of the table as a supplement to determine which eigenvalue of C_2^z the eigenstates will have when the symmetry is reduced from C_{m}^{z} to C_2^z. As can be seen from Table S1, for C_{m}^{z} symmetry, the charges of the polarization vortex can be determined up to the uncertainty of $\mathrm{n}\times\mathrm{m}$, where n is an integer which can be nonzero for higher bands[1,3]. For the discussion in the main text, the TE_1 band at Γ belongs to the B representation of C_{6v} symmetry with allowed charge -2. When the symmetry is reduced from C_{6v} to C_{2v}, the TE_1 band at Γ belongs to the B representation of C_{2v}, thus the allowed charge are 0 or -2 which correspond to the left and middle panels of Fig. 2(c), respectively. Meanwhile, since a topological charge cannot suddenly disappear, a higher charge should split into multiple integer charges or half-integer charges[4] under the symmetry reduction.

Symmetry	Representation	C_{m}^{z}	C_2^z	Allowed charges
C_{2v}	A	$+1$	$+1$	$1+2n$
	B	-1	-1	$0+2n$
C_{3v}	A	$+1$		$1+3n$
C_{4v}	A	$+1$	$+1$	$1+4n$
	B	-1	$+1$	$-1+4n$

C_{6v}	A	+1	+1	$1+6n$
	B	−1	−1	$-2+6n$

Table S1. Allowed charges for nondegenerate bands. Here, m = {2, 3, 4, 6}, n is an integer that can be nonzero for higher energy bands.

II. Theoretical models for polarization vortex and scaling rules of Q factor

Since the polarization vector, $\phi(\mathbf{k})=c_x(\mathbf{k})+ic_y(\mathbf{k})$, of the far-field radiation is a continuous function of $\mathbf{k}$ in the reciprocal space, we can expand them into Taylor series around the Γ point. Then we can derive the form of polarization vortexes by implementing the symmetry constraints to determine the coefficients of the Taylor series[5]. Taylor series can be written as:

$$c_x=\sum_{mn}a_{mn}k_+^m k_-^n\ , \tag{S1}$$

$$c_y=\sum_{mn}b_{mn}k_+^m k_-^n\ , \tag{S2}$$

Where c_x and c_y are both real, $k_+=k_x+ik_y$ and $k_-=k_x-ik_y$. If the system exhibits symmetry $\mathcal{R}$, then the polarization vector should satisfy $\phi(\mathcal{R}\mathbf{k})=\pm\mathcal{R}\phi(\mathbf{k})$, where the additional "$+/-$" comes from the symmetry/anti-symmetry representation of the plane wave basis[1].

As an example, we consider a PCS that belongs to the C_{6v} point group and the at-Γ eigenmode has the B1 representation. For the B1 representation of the C_{6v} point group, $\phi(\mathbf{k})$ fulfills the constraints $\phi(\sigma_x\mathbf{k})=-\sigma_x\phi(\mathbf{k})$ and $\phi(\sigma_y\mathbf{k})=\sigma_y\phi(\mathbf{k})$, namely,

$$\sum_{mn}(a_{mn}+ib_{mn})(-1)^{m+n}k_-^m k_+^n=\sum_{mn}(a_{mn}-ib_{mn})k_+^m k_-^n \tag{S3}$$

$$\sum_{mn}(a_{mn}+ib_{mn})k_-^m k_+^n=\sum_{mn}(a_{mn}-ib_{mn})k_+^m k_-^n \tag{S4}$$

Therefore, $a_{nm}=a_{mn}$, $b_{nm}=-b_{mn}$ and $m+n$ is an even number. As the C_6^z rotation symmetry exhibit eigenvalue of -1 for the B1 representation, $\phi(C_6^z\mathbf{k})=-C_6^z\phi(\mathbf{k})$ is satisfied, namely,

$$\sum_{mn}(a_{mn}+ib_{mn})e^{i(m-n)\pi/3}k_+^m k_-^n=-\sum_{mn}(a_{mn}+ib_{mn})e^{i\pi/3}k_+^m k_-^n\ . \tag{S5}$$

Thus, $m-n=6\mathrm{N}-2$. Meanwhile, $\phi(k_x,k_y\,|\,k_y=0)$ is related to $\phi(k_x,k_y\,|\,k_y=\sqrt{3}k_x)$ by the C_6^z rotation

operation, then

$$\sum_{mn} a_{mn} k_+^m k_-^n = -\sum_{mn} \left(\frac{1}{2} a_{mn} + \frac{\sqrt{3}}{2} b_{mn} \right) k_+^m k_-^n \, , \tag{S6}$$

which gives rise to $b_{mn} = -\frac{2\sqrt{3}}{3}\left[e^{i(n-m)\pi/3} + \frac{1}{2} \right] a_{mn}$. As a result, the polarization vector becomes

$$\phi(k_+, k_-) = 2a_{02}k_-^2 + 2a_{04}k_+^4 + 2a_{13}k^2 k_+^2 + O(k^6) \, . \tag{S7}$$

By only keeping the lowest order of the expansion, we obtain the polarization vortex as $\phi(\mathbf{k}) \propto (k_x - ik_y)^2$ for the symmetry representation B_1.

The polarization vortexes of other representations can be obtained similarilty. In this way, we can obtain polarization vectors radiated from the PCS with point groups of C_{3v}, C_{4v} and C_{6v}, as summarized in Table 2.

Symmetry	Representation	C_n^z	σ_v	σ_d	$\phi(\mathbf{k})$
C_{3v}	A_1	+1	+1		$k_x + ik_y$
	A_2	+1	−1		$k_y - ik_x$
C_{4v}	A_1	+1	+1	+1	$k_x + ik_y$
	A_2	+1	−1	−1	$k_y - ik_x$
	B_1	−1	+1	−1	$k_x - ik_y$
	B_2	−1	−1	+1	$k_y + ik_x$
C_{6v}	A_1	+1	+1	+1	$k_x + ik_y$
	A_2	+1	−1	−1	$k_y - ik_x$
	B_1	−1	+1	−1	$(k_x - ik_y)^2$
	B_2	−1	−1	+1	$i(k_x - ik_y)^2$

Table 2. Polarization vortexes for nondegenerate states. The eigenvalues of the rotation and mirror symmetry for the corresponding representations are also listed in the table for reference. Here, $\phi(\mathbf{k})$ denotes the polarization vector

distribution around a symmetry-protected BIC at the Γ point.

Polarization vortexes in the momentum space are shown in Fig. S1, where BICs are located at the center of the polarization vortexes, here the Γ point. The eigenvalues of the rotation and mirror symmetry on polarization vortexes are the same as that of the corresponding eigenvector. The topological charge of a vortex is defined as the winding number of the polarization direction counterclockwise enclosing the vortex center. As shown in Fig. S1, there is a BIC [$\phi(\mathbf{k})=0$] with a charge $q = +1$ for $\phi(\mathbf{k}) \propto k_x + ik_y$ and $\phi(\mathbf{k}) \propto k_y - ik_x$, a BIC with a charge $q = -1$ for $\phi(\mathbf{k}) \propto k_x - ik_y$ and $\phi(\mathbf{k}) \propto k_y + ik_x$, and a higher-order BIC with a charge $q = -2$ of $\phi(\mathbf{k}) \propto (k_x - ik_y)^2$ and $\phi(\mathbf{k}) \propto i(k_x - ik_y)^2$.

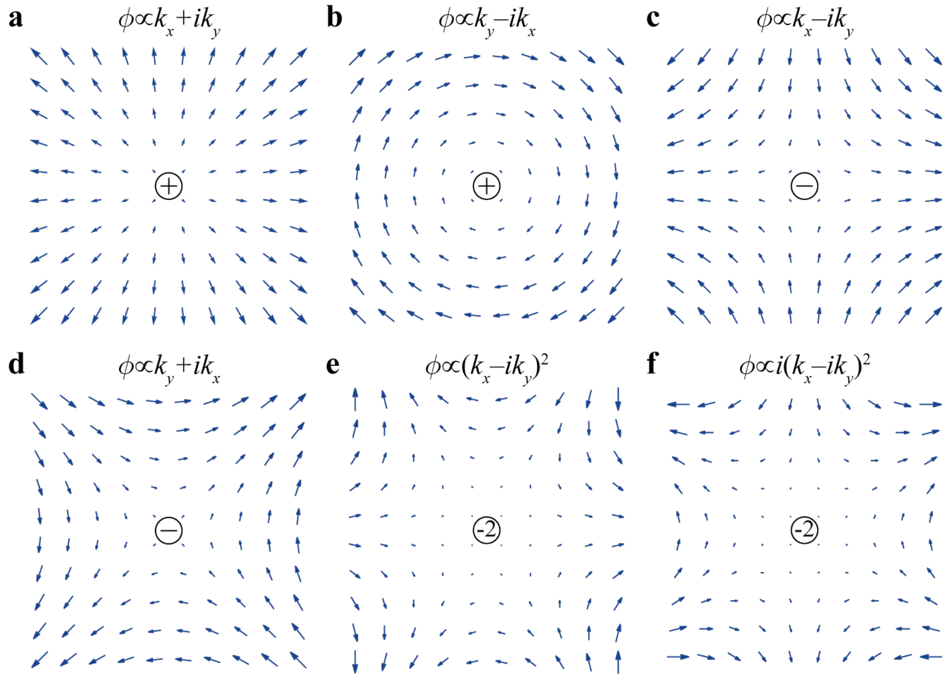

Fig. S1 Polarization vortexes in the momentum space. Polarization vectors are **a** $\phi(\mathbf{k}) \propto k_x + ik_y$, **b** $\phi(\mathbf{k}) \propto k_y - ik_x$, **c** $\phi(\mathbf{k}) \propto k_x - ik_y$, **d** $\phi(\mathbf{k}) \propto k_y + ik_x$, **e** $\phi(\mathbf{k}) \propto (k_x - ik_y)^2$ and **f** $\phi(\mathbf{k}) \propto i(k_x - ik_y)^2$. Topological charges are also provided at the centers of the vortexes.

The geometrical decay of the Q factor away from a BIC follows a scaling rule that can be derived from $Q \sim 1/|\phi(\mathbf{k})|^2$. Therefore for isolated BICs at the Γ point, the scaling rule is $Q \sim 1/k^2$ for a BIC with a charge

$q = \pm 1$, and it becomes $Q \sim 1/k^4$ for a BIC with a charge $q = -2$. More general, the scaling rule of an isolated BIC with a charge $q = \pm n$ is $Q \sim 1/k^{2n}$ (Ref. 5). We can see that the Q factor in the vicinity of an isolated BIC can be magnificently enhanced by increasing the topological charge of the BIC. Meanwhile, when another accidental BIC appears close to the isolated BIC at Γ, the scaling rule is modified. Since the accidental BICs considered here all exhibit topological charge ± 1, the scaling rule in the vicinity of each accidental BIC is $Q \sim 1/\|\mathbf{k} - \mathbf{k}_{\mathrm{BIC}}\|^2$, where $\mathbf{k}_{\mathrm{BIC}}$ represents the location of the accidental BIC and $\| \bullet \|$ represents the norm of a vector. Considering the case that the BIC at the Γ point belongs to the B_1 representation of the C_{6v} point group and there are two accidental BICs approaching from opposite directions, the scaling rule becomes $Q \sim 1/k^4 \|\mathbf{k} - \mathbf{k}_{\mathrm{BIC}}\|^2 \|\mathbf{k} + \mathbf{k}_{\mathrm{BIC}}\|^2$. In the limit $k_{\mathrm{BIC}} \to 0$, the scaling rule becomes $Q \sim 1/k^8$. Furthermore, merging a higher-order BIC with a charge $q = \pm n$ with accidental BICs can enhance the Q factor in the vicinity of the Γ point by altering the scaling rule to $Q \sim 1/k^{2n+4}$.

III. Evolution of band structures

We consider the evolution of band structures when the system symmetry is reduced by elliptic cylindrical holes. Unit cells with three different etched holes are shown in Figs. S2a-c, and the corresponding band contours of the lowest TE-like band we focus on are shown in Figs. S2d-f. Band contours are similar for the three different etched holes except for some minor distortions. As shown in Fig. S2d, when etched holes are cylindrical, band contours have rotation symmetry C_6^z and mirror symmetries σ_v and σ_d. When etched holes become elliptic cylindrical holes with the semi-major axis along the *x*-axis, there are distortions in the band contours, which reduces rotation symmetry from C_6^z to C_2^z and preserves mirror symmetries σ_x and σ_y, as shown by the band contours in Fig. S2e. When the semi-major axis rotates by θ = 10° with respect to the *x*-axis, all the mirror symmetries of the band contours are further broken with only C_2^z preserved, as shown in Fig. S2f.

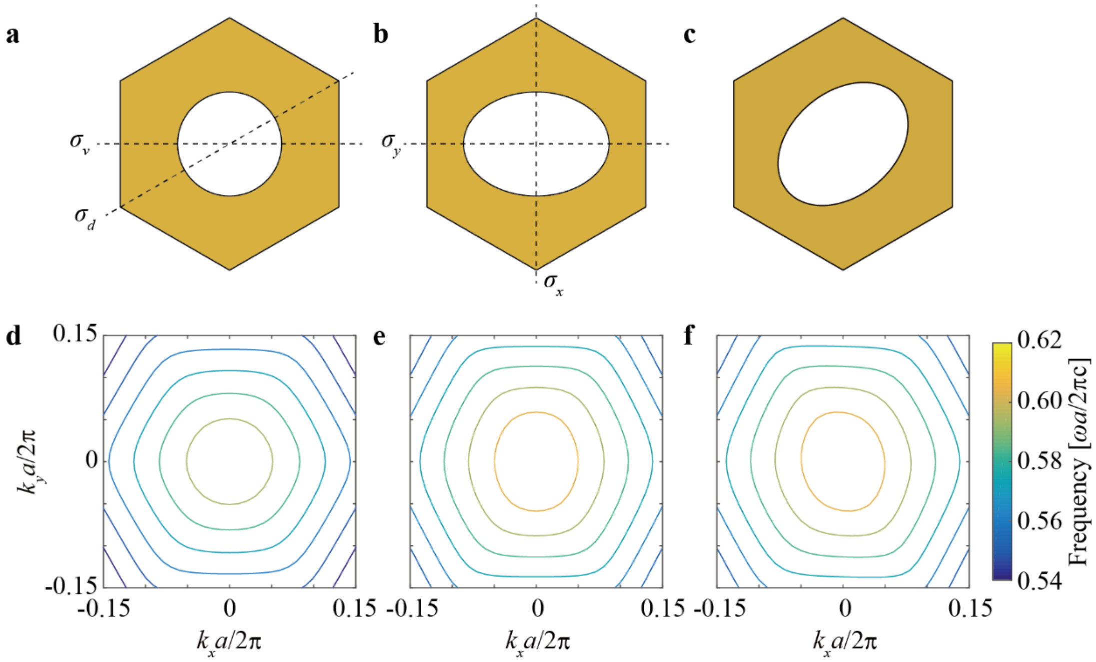

Fig. S2 Evolution of the band structures with the variation of etched holes. Schematic of unit cells with **a** a cylindrical hole, **b** an elliptic cylindrical hole with the semi-major axis along the x-axis and **c** an elliptic cylindrical hole with the semi-major axis rotated by $\theta = 10°$ with respect to the x-axis. **d-f** Simulated band contours of the lowest TE-like band in the momentum space for unit cells in **a-c**, respectively. The period is a = 336 nm. The radius of the cylindrical hole is r = 80 nm, and the semi-major axis and semi-minor axis of elliptic cylindrical holes are r_1 = 112 nm and r_2 = 80 nm, respectively. The thicknesses of the PCSs are **a** t = 340 nm and **b, c** t = 338 nm.

IV. Evolution of BICs with the variation of structural parameters

The evolution of BICs with the variation of thickness for the structure with cylindrical holes is shown in Fig. S3. The trajectories of BICs in momentum space become visible to the eye when we plot a brightness map of Q-factors. We see that the symmetry-protected BIC always exists at the Γ point, and accidental BICs are gradually tuned to the Γ point with the decrease of thickness from t = 340nm. The merging BIC is formed at t = 324.2 nm when accidental BICs are gathered at the Γ point, as shown in Fig. 1c the main text. Subsequently, accidental BICs disappear because of topological charge annihilation.

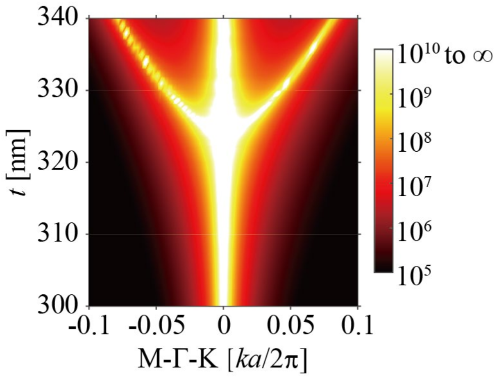

Fig. S3 The evolution of Q factors with the variation of the thickness. The evolution of Q factors for the structure with cylindrical holes. The lattice constant is a = 336 nm and the diameter of the hole is D = 160 nm.

The evolution of BICs with the variation of the aspect ratio of the elliptic cylindrical hole is shown in Fig. S4. When the semi-major axis is located along the x-axis, the symmetry-protected BIC at the Γ point is split into two BICs in the ΓM direction, as shown in Fig. S4. Here we can only see one BIC near Γ as we only show the positive half of the ΓM direction. We fix the length of the semi-minor axis at r_2 = 80 nm and increase the length of the semi-major axis so as to change the aspect ratio. We start with t = 342 nm as shown in Fig. S4a. With the increase of the semi-major axis, the split BIC is gradually tuned away from the Γ point first and then returns to the Γ point. After the two split BICs merge at the Γ point, they split once again and then shift away along the ΓK direction. Meanwhile, accidental BICs appear in both the ΓM and ΓK directions and are tuned to shift away from the Γ point with the increase of the semi-major axis. When the thickness is decreased to t = 334 nm, as shown in Fig. S4b, the split BIC can be tuned to merge with another accidental BIC along the ΓM direction. When the thickness is further decreased to t = 330 nm as shown in Fig. S4c, with the increase of the aspect ratio, the split BIC and the accidental BIC gradually approach each other to form a merging BIC and subsequently annihilate with each other. When the aspect ratio is further increased, two BICs emerge and are tuned to shift away from each other.

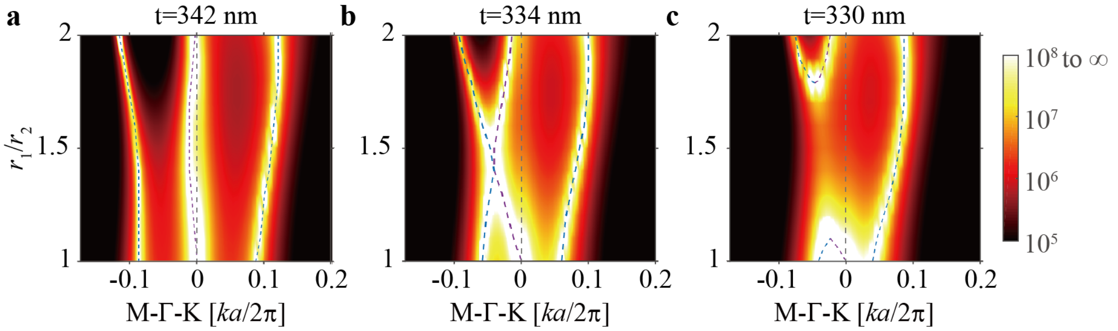

Fig. S4 The evolution of Q factors with the variation of the aspect ratio. The evolution of Q factors versus the aspect ratio at **a** t = 342 nm, **b** t = 334 nm and **c** t = 330 nm. The split BIC (magenta dashed line) is located near the

Γ point (gray dashed line), and accidental BICs (blue dashed line) are situated on both sides of Γ. In these simulations, the period is a = 336 nm, the semi-minor axis is kept at r_2 = 80 nm and the semi-major axis along the x-axis (r_1) is varied to change the aspect ratio.

Next, we study the evolution of the split BICs and accidental BICs with the variation of thickness t. When the system symmetry is reduced from C_{6v} to C_{2v}, a symmetry-protected higher-order BIC with topological charge -2 splits into two BICs in the ΓK direction at t = 350 nm. As shown in Fig. S5, with the decrease of t, the two split BICs gradually approach the Γ point and merge at t = 346.7 nm (marked by the red horizontal dashed line). After that, they are tuned to shift away from each other along the ΓM direction. At the same time, accidental BICs are tuned to approach the Γ point in both the ΓM and ΓK directions. Thus the split BICs and accidental BICs merge in the ΓM direction at t = 334.1 nm (marked by the green horizontal dashed line), and they annihilate with each other subsequently when t is further decreased.

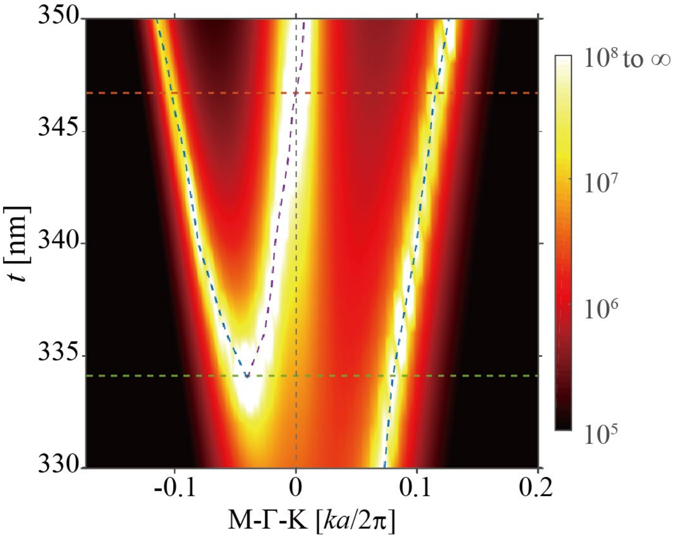

Fig. S5 The evolution of Q factors with the variation of the thickness. The evolution of Q factors for the structure with elliptic cylindrical holes. The two horizontal dashed lines (red and green) mark the thickness of the two merging BICs. The split BIC is indicated by a magenta dashed line, and accidental BICs are indicated by blue dashed lines. The period is a = 336 nm. The semi-major axis and semi-minor axis of the elliptic cylindrical hole are r_1 = 112 nm and r_2 = 80 nm, respectively.

V. Robustness of merging BICs against fabrication imperfections

We consider the influence of three typical types of fabrication errors on Q factors. In the first type, the etched hole

becomes a conical frustum with different diameters on the top and bottom, as shown in Fig. S6a. Upward and downward radiation loss cannot be eliminated simultaneously because of the disappearance of up-down mirror symmetry σ_h. In the second type, the etched hole has a tilt angle, as shown in Fig. S6b. Both σ_h and C_2^z are broken and the Q factors become finite. In the third type, the diameter of the etched hole has a deviation from the designed value, as shown in Fig. S6c. There is no symmetry broken except that off-Γ BICs deviate from the designed momenta. The distributions of Q factors with the influence of the three types of fabrication error are shown in Figs. S6d-f. We note that the symmetry-protected BIC is protected by C_6^z and does not disappear by the broken of σ_h. Compared with isolated BICs, Q factors of merging BICs maintain sufficient large in a broad wavevector range even though BICs have been broken. Similarly, merging BICs at off-Γ points are vastly superior to isolated BICs in a broad wavevector range, as shown in Fig. S7. Therefore, merging BICs are more robust in preserving high Q factors against fabrication imperfections.

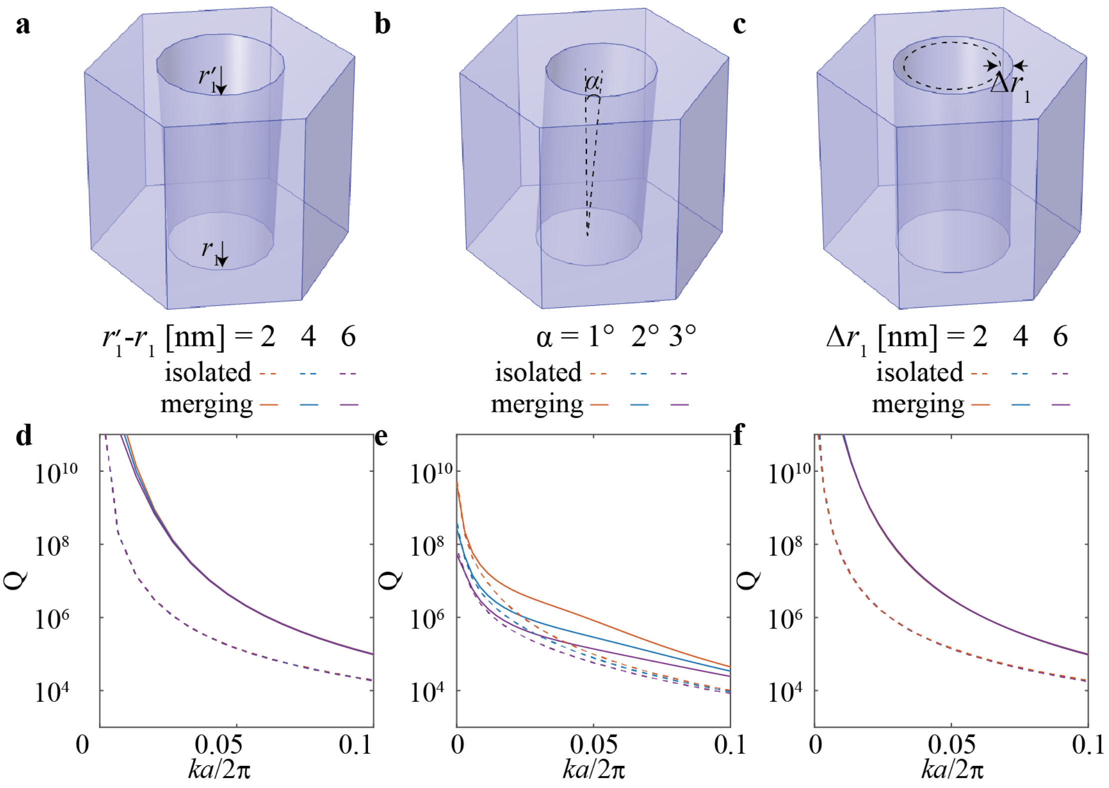

Fig. S6 Evolution of Q factors under fabrication errors for BICs at Γ. The unit cell with holes replaced by **a** conical frustums and **b** tilted holes and **c** holes with different radii. **d**, **e**, and **f** show the Q factor evolution near merging BICs (solid lines) and isolated BIC (dashed lines) under the parameters variation in **a**, **b** and **c**, respectively. Here apart from the varying parameters, other parameters are the same as Fig. 1 in the main text, $t = 324.2$ nm for the merging BIC and $t = 300$ nm for the isolated BIC.

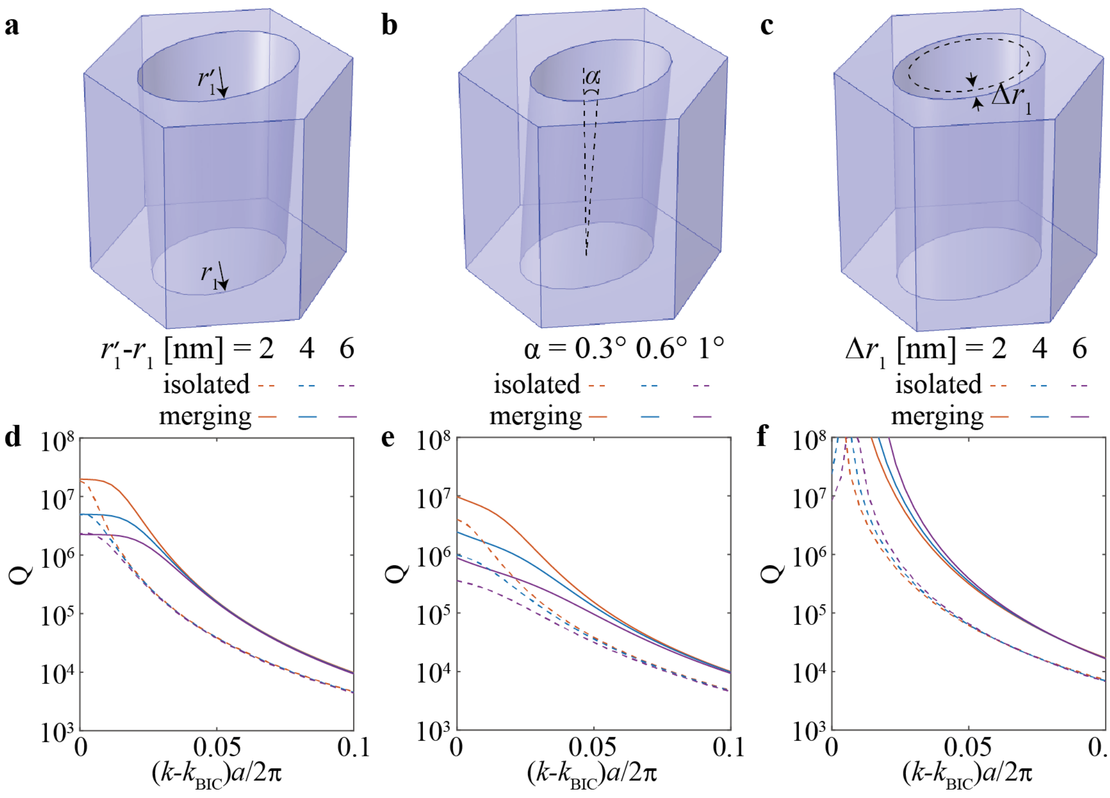

Fig. S7 Evolution of Q factors under fabrication errors for off-Γ BICs. The unit cell with holes replaced by **a** conical frustums and **b** tilted holes and **c** holes with a different semi-minor axis. **d**, **e**, and **f** show the Q factor evolution near merging BICs (solid lines) and isolated BIC (dashed lines) under the parameters variation in **a**, **b** and **c**, respectively. Here apart from the varying parameters, other parameters are the same as Fig. 3 in the main text, t = 334.1 nm for the merging BIC and t = 338 nm for the isolated BIC.

Next, we demonstrate that merging BICs are quite robust over varying thicknesses when there are three types of fabrication errors. As shown in Figs. S8 and S9, when the thickness has a deviation from the merging BIC, Q factors have a slight change and remain sufficiently large over a broad wavevector range. Therefore, the ability to overcome scattering loss is preserved.

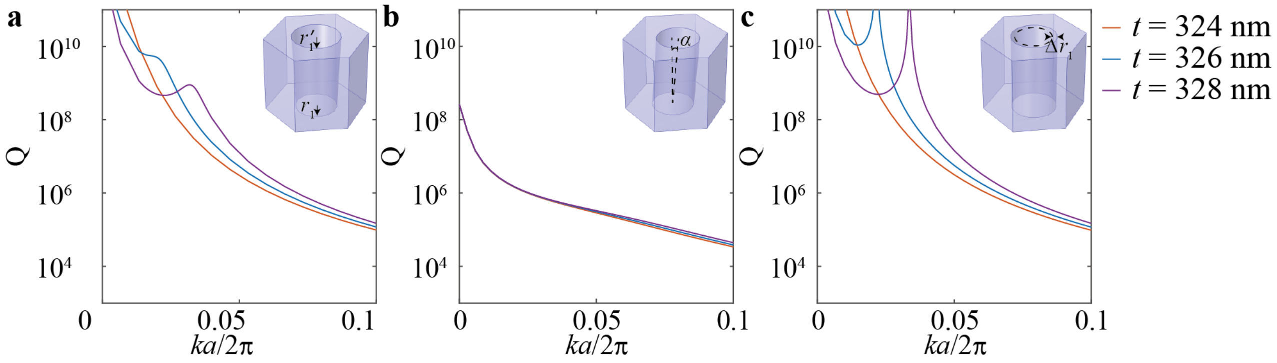

Fig. S8 Robustness over varying thicknesses for BICs at Γ. The Q factor evolution near merging BICs under the thickness variation for the etched hole with **a** conical frustums and **b** tilted holes and **c** holes with different radii. Inserts are diagrams of the unit cell. Here, $r_1' - r_1 = 4$ nm, $\alpha = 0.6°$ and $\Delta r_1 = 4$ nm.

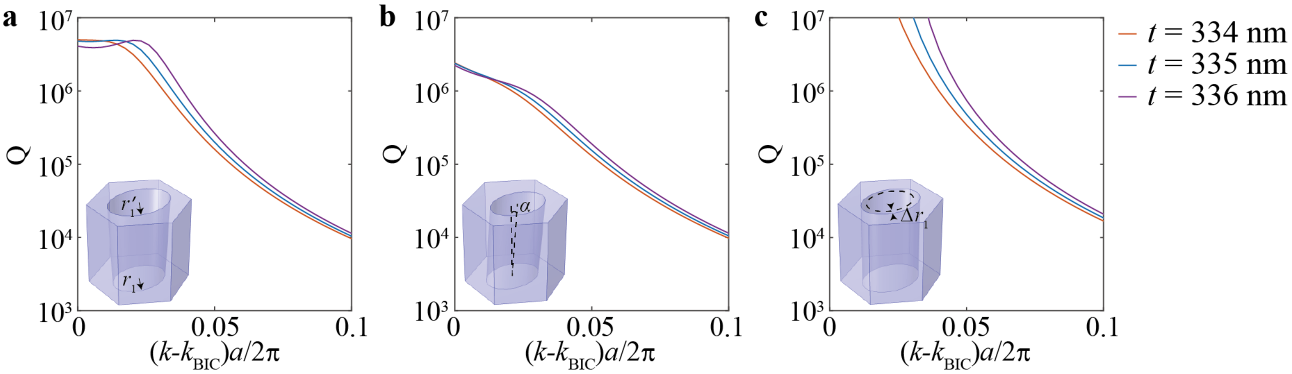

Fig. S9 Robustness over varying thicknesses for off-Γ BICs. The Q factor evolution near merging BICs under the thickness variation for the etched hole with **a** conical frustums and **b** tilted holes and **c** holes with a different semi-minor axis. Inserts are diagrams of the unit cell. Here, $r_1' - r_1 = 4$ nm, $\alpha = 0.6°$ and $\Delta r_1 = 4$ nm.

VI. Intrinsic loss of materials

The total Q factor is determined by the contribution of both radiation loss and intrinsic loss, following $\frac{1}{Q_{tot}} = \frac{1}{Q_{rad}} + \frac{1}{Q_i}$, where Q_{rad} and Q_i represent the Q factors when considering only the radiation loss and intrinsic loss, respectively. It is dominated by the intrinsic loss of materials at merging BICs, as shown in Fig. S10a. Hence materials with ultralow intrinsic loss, such as Si_3N_4 and Si, etc., are ideal choices for a high-Q cavity in the frequency range of interest. Here in our case for the merging BICs, the Q factors keep sufficiently large in a broad wavevector range even though materials have an unavoidable intrinsic loss, as shown in Figs. S10b and c.

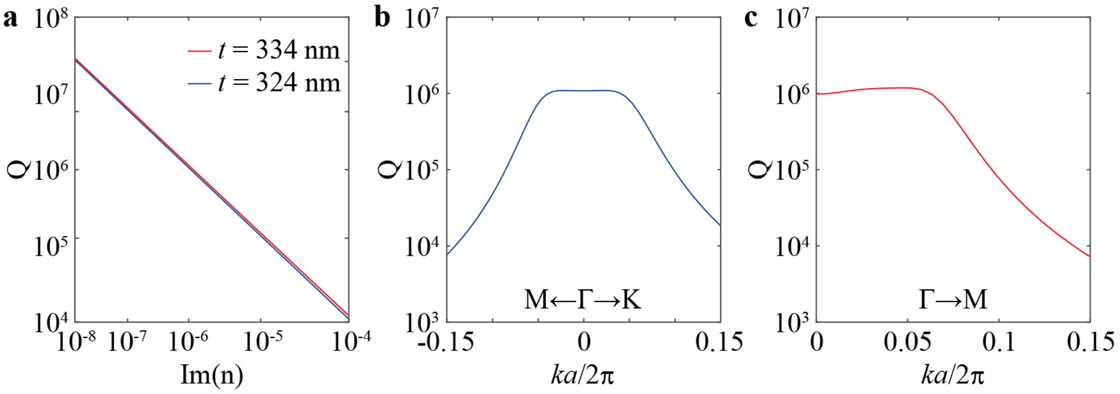

Fig. S10 The influence of intrinsic loss on merging BICs. a Numerical calculation for the evolution of Q factors

with an intrinsic loss of materials at merging BICs. The distribution of Q factors for merging BICs at the Γ point (**b**) and an off-Γ point (**c**). Im(n) = 10^{-6} in **b** and **c**. Other structural parameters keep the same as Fig. 1 and Fig. 3 in the main text for t = 324 nm and t = 334 nm, respectively.

VII. Merging BICs in degenerate bands

As shown in Fig. 1b, TE_2 and TE_3 bands are degenerate at the Γ point. The representations of the TE_2 (TE_3) band in the ΓM and ΓK directions are A (B) and B (A), respectively. Hence the degenerate state at Γ belongs to E_2 representation of the C_{6v} group and is a higher-order BIC with topological charge -2. Meanwhile, there can also be accidental BICs around the Γ point when the structural parameters are suitable. As shown in Fig. S11a, a symmetry-protected BIC at the Γ point is surrounded by 12 accidental BICs for the TE_2 band. Polarization distribution shows that the symmetry-protected BIC has a topological charge -2, accidental BICs in the ΓM direction have a charge -1, and accidental BICs in the ΓK direction have a charge +1. With the decrease of the thickness, accidental BICs are gradually tuned to the Γ point as shown in Fig. S11b, and eventually merge at t = 312.4 nm. It is interesting to note that the merging BICs on the TE_2 and TE_3 bands appear at the same t. When the thickness is further decreased, accidental BICs appear on the TE_3 band (Fig. S11c) and then move away from the Γ point as shown in Fig. S11d. Polarization distribution in Fig. S11c indicates that the accidental BICs possess -1 and +1 charges in the ΓM and ΓK directions, respectively.

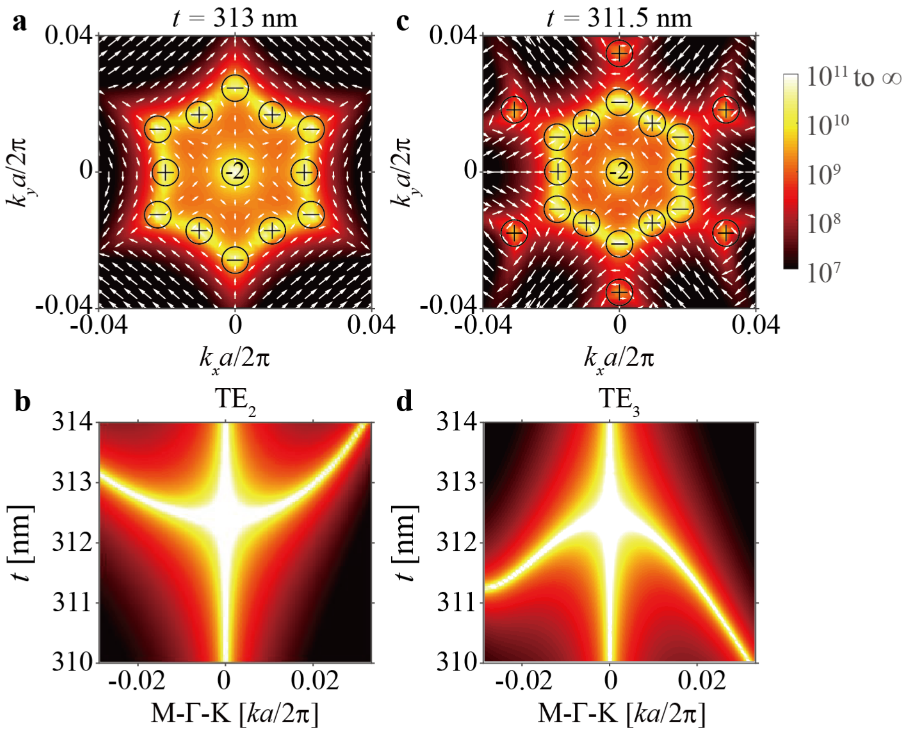

Fig. S11 Merging BICs on the degenerate bands. Simulated polarization distribution with Q factors as the background for **a** the TE_2 band at t = 313 nm and **c** the TE_3 band at t = 311.5 nm. Calculated Q factors evolution with the variation of the thickness for **b** the TE_2 band and **d** the TE_3 band. The bright curves indicate the trajectories of BICs. The parameters used are the same as Fig. 1 in the main text.

VIII. Stokes parameters S_3 of the polarization vectors

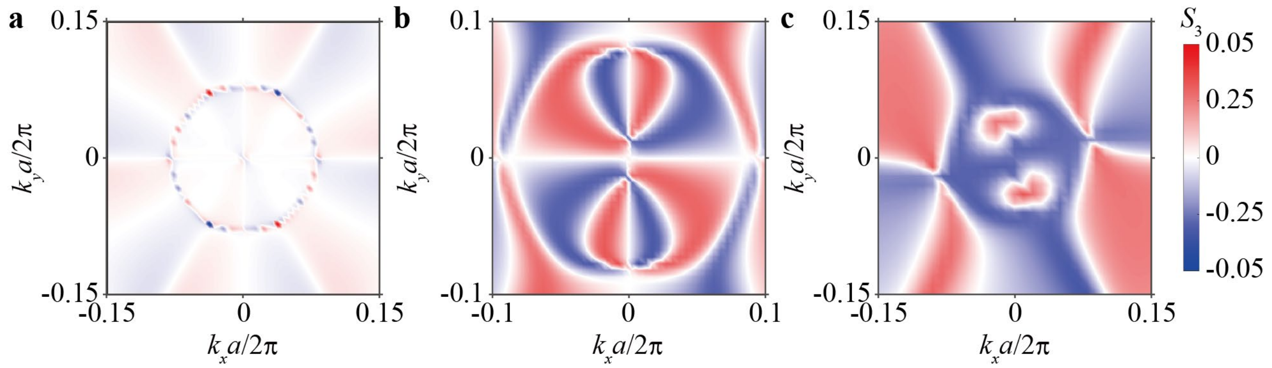

Fig. S12 Polarization ellipse parameters. a-c Simulated Stokes parameter S_3 distributions for the structures investigated in Fig. 1c, Fig. 2c and Fig. 4c in the main text, respectively. The magnitude of S_3 is small (< 0.04) within the interesting range which shows that the polarization vectors are almost linear.

References

1 Zhen, B., Hsu, C. W., Lu, L., Stone, A. D. & Soljacic, M. Topological nature of optical bound states in the continuum. *Phys. Rev. Lett.* **113**, 257401 (2014).

2 Zhang, Y. *et al.* Observation of polarization vortices in momentum space. *Phys. Rev. Lett.* **120**, 186103 (2018).

3 Iwahashi, S. *et al.* Higher-order vector beams produced by photonic-crystal lasers. *Opt. Express* **19**, 11963-11968 (2011).

4 Yoda, T. & Notomi, M. Generation and Annihilation of Topologically Protected Bound States in the Continuum and Circularly Polarized States by Symmetry Breaking. *Phys. Rev. Lett.* **125**, 053902 (2020).

5 Jin, J. *et al.* Topologically enabled ultrahigh-Q guided resonances robust to out-of-plane scattering. *Nature* **574**, 501-504 (2019).

6 Lin, Y., Feng, T., Lan, S., Liu, J. & Xu, Y. On-Chip Diffraction-Free Beam Guiding beyond the Light Cone. *Phys. Rev. Appl.* **13**, 064032 (2020).
